# Deep-sequencing transcriptome analysis of low temperature perception in a desert tree, Populus euphratica

**DOI:** 10.1186/1471-2164-15-326

**Published:** 2014-05-01

**Authors:** Jinhuan Chen, Qianqian Tian, Tao Pang, Libo Jiang, Rongling Wu, Xinli Xia, Weilun Yin

**Affiliations:** National Engineering Laboratory for Tree Breeding, Beijing, 100083 China; College of Biological Sciences and technology, Beijing Forestry University, Beijing, 100083 China; Center for Computational Biology, Beijing Forestry University, Beijing, 100083 China; Center for Statistical Genetics, The Pennsylvania State University, Hershey, PA 17033 USA

**Keywords:** Illumina/Solexa, Low temperature, *Populus euphratica*, Transcriptome

## Abstract

**Background:**

Compared with other *Populus* species, *Populus euphratica* Oliv. exhibits better tolerance to abiotic stress, especially those involving extreme temperatures. However, little is known about gene regulation and signaling pathways involved in low temperature stress responses in this species. Recent development of Illumina/Solexa-based deep-sequencing technologies has accelerated the study of global transcription profiling under specific conditions. To understand the gene network controlling low temperature perception in *P. euphratica*, we performed transcriptome sequencing using Solexa sequence analysis to generate a leaf transcriptome at a depth of 10 gigabases for each sample.

**Results:**

Using the Trinity method, 52,081,238 high-quality trimmed reads were assembled into a non-redundant set and 108,502 unigenes with an average length of 1,047 bp were generated. After performing functional annotations by aligning all-unigenes with public protein databases, 85,584 unigenes were annotated. Differentially expressed genes were investigated using the FPKM method by applying the Benjamini and Hochberg corrections. Overall, 2,858 transcripts were identified as differentially expressed unigenes in at least two samples and 131 were assigned as unigenes expressed differently in all three samples. In 4°C-treated sample and -4°C-treated sample, 1,661 and 866 differently expressed unigenes were detected at an estimated absolute log_2_-fold change of > 1, respectively. Among them, the respective number of up-regulated unigenes in C4 and F4 sample was 1,113 and 630, while the respective number of down-regulated ungenes is 548 and 236. To increase our understanding of these differentially expressed genes, we performed gene ontology enrichment and metabolic pathway enrichment analyses. A large number of early cold (below or above freezing temperature)-responsive genes were identified, suggesting that a multitude of transcriptional cascades function in cold perception. Analyses of multiple cold-responsive genes, transcription factors, and some key transduction components involved in ABA and calcium signaling revealed their potential function in low temperature responses in *P. euphratica*.

**Conclusions:**

Our results provide a global transcriptome picture of *P. euphratica* under low temperature stress. The potential cold stress related transcripts identified in this study provide valuable information for further understanding the molecular mechanisms of low temperature perception in *P. euphratica*.

**Electronic supplementary material:**

The online version of this article (doi:10.1186/1471-2164-15-326) contains supplementary material, which is available to authorized users.

## Background

Low temperature has strong influence on the geographical distribution, growing season, quality and yield of plants. Previous reports have shown that plants may develop acquired freezing tolerance after exposure to non-freezing low temperatures [1, 2]. Plant cells cope with cold stress by regulating the expression of transcription factors and effectors during non-freezing low temperatures [3]. However, the transcriptome-level changes that underlie perception of temperatures below zero, which may be related to the ability to survive under extremely low temperatures [4], is poorly understood comparing that with cold sensing above freezing temperature.

A variety of cold-responsive (COR) genes that are under the control of some key transcription factors (TFs) are thought to be involved in cold sensing [5]. For example, the well characterized TF DREB1/CBF can regulate a subset of COR genes by binding the DRE/CRT *cis*-elements in promoter regions of COR genes [6–8]. By studying the DREB1/CBF network pathway, the roles of cellular or environmental factors, e.g. calcium, light, and circadian rhythm, are revealed in cold acclimation [1, 9]. The DREB1/CBF pathway in chilling response seemed well characterized in some plants, and its regulon has been identified in *Brassica napus*, rice, and poplar. However, the molecular mechanisms of cold-acclimation response are not well understood at the whole genome or transcriptome level since only 12% of cold responsive genes are members of the CBF regulon [10, 11]. It remains to be answered whether low temperature perception occurs below freezing temperature, and if so, whether it occurs by a similar molecular mechanism as above freezing temperature.

*Populus euphratica* Oliv. is naturally distributed in semiarid areas and plays an important role in maintaining local arid ecosystems [12]. They distinguish themselves considerably from other species by growing in deserts with extremely salty soil and wide environmental temperature ranges. Thus, *P. euphratica* has been widely considered as a model species for elucidating abiotic resistance mechanisms in trees [13–17]. Screening for cold responsive genes in *P. euphratica* can be a useful approach to understand the responses of woody plants to low temperatures and can also help elucidate the difference in cold perception between below- and above- freezing temperatures.

Recently, the development of Illumina/Solexa-based deep-sequencing technologies has made it possible to capture an unbiased view of the RNA transcript profile of a species under a given condition at the whole genome level [18]. Using this method, ESTs and numerous novel transcripts have been discovered in a tissue-specific manner [19, 20]. In the current study, we sought to identify genes linked to low temperature (below or above zero) perception and to explore the regulatory and sensory mechanisms involved in low temperature response processes by performing de novo assembly of the *P. euphratica* transcriptome using Solexa data. Two-year-old plants were subjected to temperatures of 4°C and a further drop to -4°C to conduct comprehensive analysis of transcriptional responses. The acquired information may facilitate attempts to elucidate response mechanisms of this species to chilling stress and will help in the development of strategies for improving of freezing tolerance in trees.

## Results and discussion

### Reads assembly and poplar databses alignment

**Figure 1:**
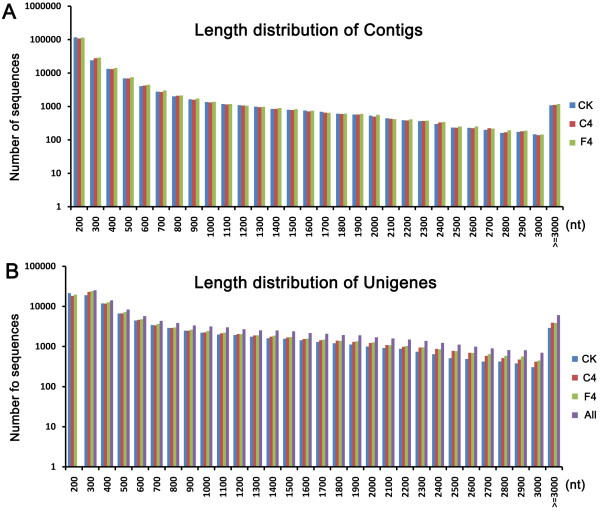
**Overview of assembly by Trinity. (A)** Length frequency distribution of contigs obtained from *de novo* assembly of high-quality clean “reads”. **(B)** Length frequency distribution of unigenes produced by contig joining.

Three cDNA libraries were generated with mRNA from control (22°C), 4°C- or -4°C-treated *P. euphratica* plants and sequenced by Illumina deep-sequencing. After removing adapters, low-quality sequences, and ambiguous reads, a total of 132 million, 135 million, and 134 million clean reads with a mean length of 90 bp were generated in the control (CK), 4°C-treated sample (C4), and -4°C-treated sample (F4), respectively (Table 1). The raw data were deposited in the NCBI Sequence Read Archive (SRA) under the accession number SRP026075. The total length of the reads was >30 gigabases (Gb), equivalent to ~75-fold coverage of a *P. trichocarpa* genome. All trimmed reads were de novo assembled into contigs by the Trinity method [21]. The average contig size exceeded 320 bp in each of the three libraries (Figure 1A). Using paired-end information, the contigs were joined into assembled unigenes. Over 80% reads could be mapped back to the assembled transcripts, indicating a high quality of assembly (Additional file 1). Finally, 108,502 unigenes with an average length of 1,047 bp and N50 of 1,821 bp were assembled (Table 1). All unigenes were longer than 200 bp and 11.34% (12,309) of them were longer than 1,000 bp (Figure 1B).

**Table 1:** **Overview of the sequencing and assembly**

Sequences	CK	C4	F4
Total Raw Reads	139,967,622	144,120,358	146,769,184
Total Clean Reads	132,575,472	135,458,532	134,977,754
Total Clean Nucleotides (nt)	11,931,792,480	12,191,267,880	12,147,997,860
Q20 percentage	97.66%	97.41%	97.63%
N percentage	0.00%	0.00%	0.00%
Total Number of contig	185,758	180,385	190,969
Total Length of contig (nt)	59,420,994	58,876,674	62,206,357
Mean Length of contig (nt)	320	326	326
N50 of contig	524	530	523
Total Number of unigne	98,171	103,563	108,769
Total Length of unigne (nt)	73,038,351	83,985,593	86,583,871
Mean Length of unigne (nt)	744	811	796
N50 of unigene	1462	1631	1601

To estimate the representation of unigenes in the collection, all unique sequences generated from different assemblages were subjected to a BLAST comparison to compare EST collections from a variety of *Populus* species. The results indicated that our assemblages covered most *P. euphratica* transcripts (Additional file 2). By performing BLASTx against the *Populus trichocarpa* v3 dataset with an E-value of 1.0E-5 as threshold, 83,618 ESTs were assigned with an identity score ≥ 75%, covering 77.07% of assembled unigenes (Additional file 3). Of these unigenes, 79, 389 (97.3%) members shared >90% identity with their homologs from *P. trichocarpa*. Meanwhile, 3,6559 homologs (>80%) of *P.* trichocarpa v3 gene models have been sequenced. All these results indicate that our RNA-sequencing data has high contiguity, coverage, and could be used for further analyses.

### Functional annotation and classification of the unigenes

**Figure 2:**
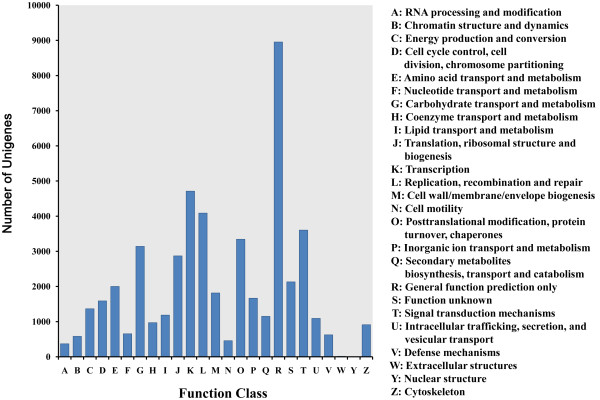
**COG functional classification of the** ***P. euphratica*** **transcriptome.** 49,291 unigenes with significant homologies in the COG database (E-value < 1.0 E-5) were classified into 25 COG categories. The capital letters in x-axis indicates the COG categories as listed on the right of the histogram, and the y-axis indicates the number of unigenes.

**Figure 3:**
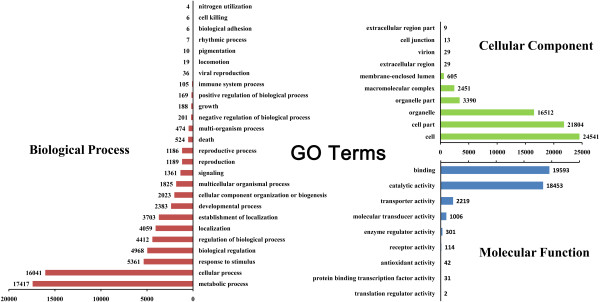
**Function classifications of GO terms of all** ***P. euphratica*** **transcripts.** Based on highscore BLASTx matches in the NR plant proteins database, *P. euphratica* unigenes were classified into three main GO categories and 31 sub-categories. The left y-axis indicates the percentage of a specific category of genes in each main category. The right y-axis indicates the number of genes in the same category.

Using the best hits found by BLASTx or BLASTn with an E-value of < 1.0E-5, all of the unigenes were annotated according to the public databases including non-redundant protein (NR) database, non-redundant nucleotide (NT) database, SwissProt, Kyoto Encyclopedia of Genes and Genomes (KEGG) database and Clusters of Orthologous Groups (COG) database on the basis of similarities. The number of unigenes annotated with each database is summarized (Additional file 4). Of the 108,502 high-quality unique sequences, 85,584 (78.88%) unigenes had at least one significant match to an existing gene model in the BLAST searches (Additional file 4). By performing BLASTx against the NR database with an E-value cut-off of 1.0E-5, 71,428 BLASTx hits were obtained, covering 83.5% of the annotated unigenes. Within the *P. euphratica* unigene set, 49,291 (45.43%) unigenes were categorized (E-value < 1.0E-5) in 25 COG clusters (Figure 2). The five largest categories were: 1) general function predictions only (18.2%), 2) transcription (9.6%), 3) replication, recombination and repair (8.3%), 4) signal transduction mechanisms (7.3%) and 5) post-translational modification, protein turnover, chaperones (6.8%). Classification of Gene ontology (GO) terms was performed according to the NR annotation using the Blast2GO software [2]. In the category of biological process, the largest groups were cellular process, metabolic process, response to stimulus, and biological regulation (Figure 3). As for the molecular function category, unigenes with binding and catalytic activity formed the largest groups.

To obtain a better understanding of the biological functions of the unigenes, a KEGG pathway-based analysis was also performed. Based on a comparison against the KEGG database using BLASTx with an E-value cutoff of <1.0E-5, 39,313 (36.23%) of the 108,502 unigenes had significant matches in the database and were assigned to 127 KEGG pathways. Of the 8,220 metabolism pathway unigenes, 2,726 were involved in plant hormone signal transduction pathways, including tryptophan metabolism, zeatin biosynthesis, diterpenoid biosynthesis, carotenoid biosynthesis, cysteine and methionine metabolism, brassinosteroid biosynthesis, α-Linolenic acid metabolism, and phenylalanine metabolism.

The three samples had 68 members in common when the 100 most abundant transcripts were compared (Additional file 5). The 23 unique members highly expressed in the control were involved in auxin signaling, cell division, and biogenesis. In contrast, the 19 unique members highly expressed in the C4 sample were stress (e.g., arginine decarboxylase, and dehydration) -induced genes. The 28 unique members highly expressed in the F4 sample were also stress-related genes, e.g., the glucanase, zinc finger protein, and E3 ubiquitin-protein ligase genes. These results indicate that our data are reliable.

### Protein coding sequence prediction

**Figure 4:**
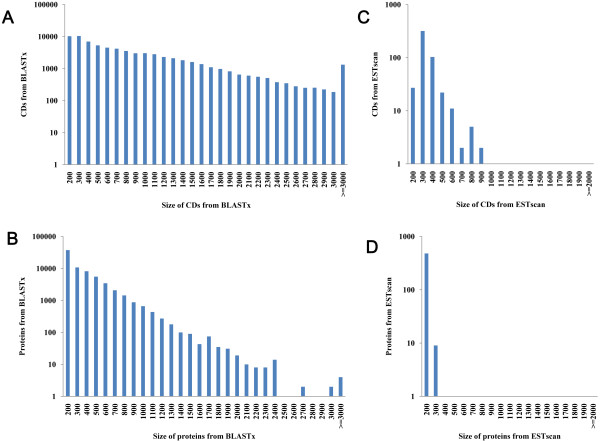
**Transcriptome coding sequence (CDS) predicted by BLASTx and ESTScan. (A)** The length distribution of CDs using BLASTx. **(B)** The length distribution of proteins using BLASTx. **(C)** The length distribution of CDs using ESTscan. **(D)** The length distribution of proteins using ESTscan.

Unigenes were aligned by BLASTx (E-value < 1.0E-5) against the NR, Swiss-Prot, KEGG, and COG protein databases in that order. Unigenes aligned to a high priority database were not aligned to databases of lower priority. The process ended when all alignments had been performed. The correct reading frame of the nucleotide sequences (5’-3’direction) of unigenes was defined by the highest rank in the BLAST results, and the corresponding protein sequences were obtained from the standard codon table. Unigenes that could not be aligned to any database were scanned with ESTScan [22] to produce the nucleotide and amino acid sequences of the predicted region. In total, 71,559 unigene coding sequences (CDSs) were generated by the BLASTx protein database searches described above. Of these unigenes with CDS sequences, the majority (44,005 members, occupied 61.5%) were over 500 bp and 23,479 were over 1, 000 bp in length (Figure 4A-B). Using the ESTscan program, we assigned another 489 unigene CDSs that could not be aligned to above databases (Figure 4C-D). The length frequency distributions of these unigene CDSs and their corresponding amino acid sequences are given (Figure 4).

### Differentially expressed gene among three samples

We measured gene expression levels based on fragments per kilobase of exon model per million mapped reads (FPKM). After applying the chi-square test and Benjamini-Hochberg multiple testing corrections using R program among three samples simultaneously, we identified 2,858 genes as reliable DEGs in at least two samples (assigned as either DEGs) regardless of fold change (Additional file 6). Of these DEGs, 131 were expressed differentially in all three samples (assigned as all-DEGs, Additional file 7). Given a standard at an estimated absolute log_2_-fold change of >1, the respective DEGs of CK vs. C4, CK vs. F4 and C4 vs. F4 were 1,661, 866, and 1,161 (Additional files 8, 9, 10). The number of up-regulated unigenes in C4 and F4 samples was 1,113 and 630, respectively.

To accurately identify DEGs, we selected the 50 most significantly up-regulated transcripts that could be well-annotated by poplar database or NR database. As a result, those coding for the chlorophyll a/b binding protein (e.g., Unigene50811, CL12828.Contig3, Unigene50363, and Unigene55266), rubisco activase (CL4046.Contig4, Unigene50527, and Unigene55538), AP2/ERF transcription factors (Unigene26311,Unigene22719, CL9386.Contig2, Unigene18453, and CL9876.Contig3), and some other transcription factors (CL1721.Contig8, and Unigene27837) were the most up-regulated interpretable transcripts in C4 sample (Additional file 11). As for the top 50 up-regulated transcripts in the F4 sample (Additional file 12), the annotated transcripts focused on transcription factors (DREB1 transcription factors e.g. unigene26567 and unigene26311; WRKY transcription factor Unigene18620) and xyloglucan endotransglycosylases (Unigene19292, Unigene14078 and CL29.Contig1).

**Figure 5:**
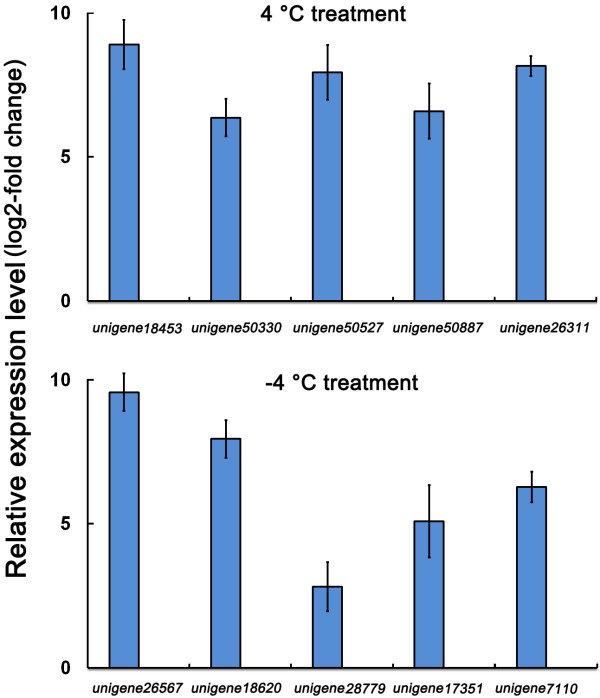
**Expression analyses of 10 DEGs by qPCR.** qPCR was performed on 10 members randomly selected from up-regulated gene lists of the C4 or F4 sample.

Although Illumina sequencing is a highly efficient method for DEG screening, false positives still occur because of the sensitivity of this technology to templates present in DNA samples [23]. Thus, we validated the RNA sequencing data by performing qPCR analysis on 10 transcripts randomly selected from the up-regulated gene list. The qPCR results indicate that all of these DEGs exhibited similar expression kinetics to those obtained from the RNA sequencing analysis (Figure 5). These results support the validity of the method used for determining DEGs from the RNA sequencing analysis.

### Gene ontology and pathway enrichment analyses of differentially expressed unigenes

All DEGs were mapped to each term of the Gene Ontology database (http://www.geneontology.org/, release data: Aug 1st, 2012) and the gene numbers were calculated from each GO term. Using a hypergeometric test, we identified the significantly enriched GO terms of DEGs compared to the genomic background (*p* ≤ 0.05, after Bonferroni correction). In the category of biological processes, three Go terms including “response to stress”, “response to stimulus” and “response to carbohydrate stimulus” are enriched (*p* ≤ 0.05, after bonferroni correction) after 4°C and -4°C treatments (Table 2), suggesting that genes in these processes may play important roles in low temperature perception. Additionally, “carbon fixation process”, “glucan metabolic process” and “macromolecule metabolic regulation processes” are also enriched for DEGs in C4 (Table 2), indicating that genes related to these processes may also participate in cold sensing. A close inspection referred to “response to stimulus” category indicated that “response to hormone stimulus” and “response to abiotic stimulus” were two over-presented subcategories (data not shown), suggesting our low temperature treatment may have caused an efficient abiotic stress and have activated some hormone response process. Furthermore, DEGs with “protein binding” and “protein modification” subcategories were also over-presented in both samples, indicating that comprehensive changes had taken place in cells in response to low temperature stress. We further performed Go enrichment analysis for genes that differentially expressed in all of three samples and the results indicated that those involved in gene expression regulation, macromolecule metabolic process regulation, and abiotic stimulus response were enriched. As for the category of “molecular function”, DEGs with “structural molecule activity” was the only common group that over-presented after 4°C and -4°C treatments (Table 2).

**Table 2:** **Over-representative GO terms of DEGs in low temperature stressed** ***P. euphratica*** **GO ID**

	Categoray	Description	Ccorrected ***p***-value
**DEGs in 4°C treatment**			
6950	P	response to stress	1.50E-08
10033	P	response to organic substance	5.20E-08
50896	P	response to stimulus	6.60E-08
9743	P	response to carbohydrate stimulus	5.20E-07
9719	P	response to endogenous stimulus	8.90E-07
15977	P	carbon fixation	2.30E-06
9725	P	response to hormone stimulus	4.10E-06
71704	P	organic substance metabolic process	4.90E-06
5982	P	starch metabolic process	9.80E-06
6952	P	defense response	3.80E-05
42221	P	response to chemical stimulus	4.50E-05
44042	P	glucan metabolic process	1.20E-04
19222	P	regulation of metabolic process	1.30E-04
60255	P	regulation of macromolecule metabolic process	2.90E-04
16160	F	amylase activity	1.20E-05
16758	F	transferase activity, transferring hexosyl groups	1.30E-04
5198	F	structural molecule activity	2.00E-04
16831	F	carboxy-lyase activity	3.20E-04
34357	C	photosynthetic membrane	9.50E-06
9521	C	photosystem	9.10E-06
**DEGs in -4°C treatment**			
6950	P	response to stress	9.40E-06
50896	P	response to stimulus	3.90E-05
9743	P	response to carbohydrate stimulus	3.30E-05
5198	F	structural molecule activity	7.20E-12
4857	F	enzyme inhibitor activity	3.70E-05
9521	C	photosystem	4.80E-04
34357	C	photosynthetic membrane	6.00E-04
32991	C	macromolecular complex	1.00E-03
30312	C	external encapsulating structure	1.60E-03
**All-DEGs**			
10468	P	regulation of gene expression	8.5-05
6350	P	transcription	9.9-05
60255	P	regulation of macromolecule metabolic process	1.7-04
19222	P	regulation of metabolic process	2.4-04
9628	P	response to abiotic stimulus	1.4-03
34645	P	cellular macromolecule biosynthetic process	1.9-03
9059	P	macromolecule biosynthetic process	2.2-03
50789	P	regulation of biological process	2.8-03

By performing the KEGG pathway analyses, we identified twelve pathways that changed significantly (*q* ≤ 0.05) under 4°C treatment, including the members involved in carbohydrate, energy, vitamin, hormone, and nitrogen metabolism (Additional file 13). “Plant pathogen interaction”, “hormone signal transduction”, and “biosynthesis of unsaturated fatty” pathways had the top three most differentially expressed unigene numbers and thus seem to play important roles in low temperature perception above freezing point. As for -4°C treatment, only 3 pathways changed significantly (Additional file 13). “Plant pathogen interaction” was assigned as a major pathway that changed significantly in both treated groups, indicating that low temperature stress response signal network may overlap with plant-pathogen interactions signals in *P. euphratica.* This is a notable finding considering that little is known about the overlap in signal transduction between abiotic and biotic stresses. Additionally, the transcripts of all of unsaturated fatty acid pathway genes increased significantly in the C4 sample. This result is in accordance with previous reports that plants undergoing low temperature stress preferentially accumulate poly-unsaturated and unsaturated fatty acids, which enhance low temperature tolerance under chilling conditions [24, 25].

### Transcription factors responding to low temperature stress

Transcription factors play crucial roles in the regulation of target gene expression via specific binding to *cis*-acting elements in their promoters [26]. Many of the COR genes contain *cis*-elements, such as dehydration-responsive elements/C-repeat elements (DRE/CRT, A/GCCGAC) and myeloblastosis (MYB, C/TAACNA/G) [27, 28] in their promoters that can be regulated by DREB and MYB transcription factors. Analysis of these transcription factors could provide useful information on the complex regulatory networks involved in *P. euphratica* cold stress responses.

Changes in the expression of transcription factors occurred both after 4°C and -4°C treatments (Table 3). The AP2/ERF transcription factors were overrepresented ( log_2_-fold change > 1) in both treated samples. This family contains 24 and 22 up-regulated members in the C4 and F4 samples, respectively (Table 3), indicating its important role in low temperature stress responses. The AP2/ERF transcription factors have been subdivided into five subfamilies including AP2 subfamily, DREB subfamily, ERF subfamily, RAV subfamily and others. Some RAP homologs (e.g. unigene16978 and CL5587.contig2) and ERF homologs (e.g. unigene8840, CL4762.contig1, CL13298.contig1), which were seldom studied in cold sensing were up-regulated in both C4 and F4 samples, indicating the potential function of these subfamilies in cold response. As a group of DREB subfamily, CBF/DREB1 was found to be expressed specifically under cold stress but not under normal growth conditions. Here, several *DREB1/CBF*-like unigenes changed their expression significantly after low temperature treatments. The transcripts of two CBF4/DREB1D homologs, Unigene26311 and Unigene22719, both increased over 11-fold after both treatments (Additional files 11, 12). However, no *Arabidopsis* CBF2 homologs were found up-regulated in the *P. euphratica* transcriptome. Thus, our results not only indicate a key role of the CBF/DREB1 transcription factors in low temperature responses but also suggest that the CBF/DREB1 transcriptional activation mechanism of *P. euphratica* is not necessarily the same as that of *Arabidopsis*.

**Table 3:** **Distribution of differentially expressed transcription factors**

Category	Total	C4		F4	
		Up -regulated	Down -regulated	Up -regulated	Down -regulated
B3	73	1	0	2	0
BES1	17	1	0	0	0
bHLH	243	4	0	0	0
C2H2	105	4	1	5	0
C3H	112	3	3	3	1
CO-like	19	1	2	1	0
CPP	10	1	0	0	0
Dof	54	3	1	1	0
ERF	183	24	4	22	3
G2-like	103	1	0	0	0
GATA	77	3	1	2	0
GRAS	119	5	0	4	0
HD-ZIP	155	0	3	0	1
NAC	150	9	0	5	0
NF-YB	25	0	1	0	0
NF-YC	40	0	1	0	0
RAV	4	1	0	2	0
S1Fa-like	3	0	0	0	1
SBP	44	2	0	0	0
TCP	37	0	1	0	0
Trihelix	78	1	0	0	0
WOX	64	0	1	0	1
WRKY	206	20	0	12	0

Each transcription factor contains known DNA-binding domains defined by the Pfam database.

Previous studies have shown that not all cold-regulated gene expression is under the direct control of the CBF/DREB family [11, 29]. Besides the AP2/ERF family, it is likely that the WRKY and NAC transcription factors also play important roles in the transcriptional regulation of genes in early cold response in *P. euphratica* because they were overrepresented in the up-regulated gene list (log_2_-fold change > 1). In the WRKY family, 20 and 12 members were up-regulated in the C4 and F4 groups, respectively (Table 3). In comparison, none was found down-regulated in the respective groups. In the NAC transcription factor family, the transcripts of 9 and 5 members were up-regulated, while none was found down-regulated in both treated samples. Evidence that the WRKY and NAC transcription factor gene families may play important roles in the regulation of transcriptional reprogramming associated with cold stress responses is incremental [30–33]. The role of several WRKY genes, such as AtWRKY25, AtWRKY33, HvWRKY38, and GmWRKY21 [34–36], and several NAC genes, such as TaNAC2, SsNAC23, and OsNAC5 [30, 37, 38], in plant cold response has been established. The overrepresentation (log_2_-fold change > 1, FDR ≤ 0.05) of these two transcription factor families in the up-regulated gene list may indicate their importance in *P. euphratica* cold perception.

It is notable that transcription factors occupied about 20% members (9 out of 50 in C4, and 11 out of 50 in F4) in top 50 up-regulated gene lists (Additional files 11, 12). However, our analysis also showed that some transcription factors were not always up-regulated. For example, HD-ZIP transcription factors from both samples were found down-regulated. Some down-regulated members were also identified in C3H and bHLH families. According to previous reports, the bHLH family is involved in long term responses to low temperatures [39]. Some ICE1-like bHLH transcription factors may be involved in CBF regulation [40]. Here, several members of this transcription factor family have altered their expression in C4 sample but not in F4 sample. It seems likely that some bHLH transcription factors may play important roles in response of low temperature stress above freezing point. These results imply that the mechanism by which these genes mediate low temperature perception is complex.

### Photosystem response to low temperature

The eventual effect of an abiotic stress on plant growth not only depends on the extent of the damage, but also on the capacity for recovery after the damage occurs [41]. Cold-tolerant plants usually show a lower decrease in the rates of photosynthetic electron transport and photosynthetic carbon metabolism [42, 43]. These changes facilitate better recovery from chilling stress in these plants compared with cold-sensitive genotypes [44].

GO enrichment results showed that “carbon fixation” was one of the main biological processes after 4°C treatment. It was consistent with the over-representation of numbers of Ribulose bisphosphate carboxylase/oxygenase (Rubisco) genes [45] listed in the top 50 up-regulated unigenes of C4 sample. However, none was found in the top 50 up-regulated unigenes of F4 sample. Thus, we conclude that the expression elevation of *Rubisco* genes under low temperature below freezing point may be part of an adaptive mechanism that promotes *P. euphratica* survival.

Chilling-induced photoinhibition and subsequent recovery has been studied in maize. No changes or even increases in the photosystem I (PSI) activity have been observed [46]. As for the photosystem II (PSII) system, an increase in proportion of its inactive centers and a lower capacity for repair of its damaged centers were observed and these changes were assumed to have led to persistent depression of photosynthetic efficiency [47]. According to our data, a number of genes encoding photosystem I or II protein complexes exhibited up-regulation under 4°C treatment, while only a few of them up-regulated after -4°C treatment. Differential expression patterns of photosystem genes in the C4 and F4 samples suggest that photosystem could percept low temperature above but not below freezing point. This result is consistent with the findings that the photosystm of cold-acclimated plants was less affected than that of the nonacclimated plants [48].

### ABA signal transduction components in P. euphratica cold response

Current evidence suggests that multiple mechanisms are involved in activating the cold-acclimation response. Both ABA-dependent and -independent pathways could regulate cold-responsive genes. ABA is an important stress hormone that mediates abiotic stress responses in plants [1]. Plants grown under cold stress may experience altered hormone homeostasis and/or signal transduction [9]. Chen et al. [49] found that ABA levels increased in cold-treated plants. The exogenous application of ABA at normal temperature has been found to enhance the freezing tolerance and thus aid in cold-acclimation in several plant species. However, whether ABA has a fundamental role in cold sensing and cold responsive network is unresolved.

To investigate whether ABA is involved in cold perception, we first investigated expression patterns of ABA biosynthesis genes annotated by NR or poplar transcript annotation. Sharp increase in fold-change of two unigenes (CL5772.Contig2 and CL5772.Contig1) encoding the key rate-limiting enzymes of ABA synthesis (namely, 9-cis epoxycarotenoid dioxygenase or NCED1) were observed in both samples [50]. These results are consistent with those obtained in *Arabidopsis* in previous studies, suggesting that ABA biosynthesis may be involved in cold response [51, 52]. Recently, clade A protein phosphatases type 2C (PP2Cs) function as key negative regulators of ABA signaling by interacting with ABA receptors has been established [53, 54]. Here, all candidate *PP2C* genes identified in C4 and F4 samples showed an up-regulated expression pattern (Additional files 8, 9). Taken together, our results suggest ABA may function as an important mediator of low temperature perception and promoter of chilling and freezing tolerance in *P. euphratica*.

### Calcium signal transduction components in P. euphratica cold response

As a second universal messenger involved in abiotic stress responses in plants, there is increasing evidence that calcium functions as an important messenger in a low temperature signal transduction pathway [1, 55]. In *Arabidopsis*, cytoplasmic calcium levels increase rapidly in response to low temperatures, largely due to an influx of calcium from extracellular stores [56]. Calcium signals are perceived by calcium sensors that relay the signals into downstream targets such as phosphorylation cascades and gene expression regulation [57]. Three major families of Ca^2+^ sensors function in abiotic stress responses in higher plants: calmodulins (CaMs) and CaM-like proteins, calcineurin B-like (CBL) proteins, and calcium-dependent protein kinases (CDPK) [58, 59]. CaMs and CBLs are small proteins that transmit the Ca^2+^ signal by interacting with target proteins and regulating their activity. CDPKs are monomeric proteins containing a CaM-like domain with four EF-hand motifs [60].

To investigate whether these Ca^2+^ sensor are involved in cold perception of *P. euphratica*, we searched for calcium related genes in differentially expressed gene list. As a result, we have found 41 members including CDPK homologs, calmodulin (CaM)-binding protein genes, CBL-interacting protein kinases (CIPKs), and Ca^2+^-ATPase up-regulated (Additional file 8). Only 3 of them showed down-regulated after 4°C treatment. As for -4°C treatment, 19 out of 21 calcium related genes showed up-regulated while only 2 were down-regulated (Additional file 9). These results are consistent with previous studies on *Arabidopsis* and rice showing that low temperature enhances the gene expression of Ca^2+^ sensors such as Ca^2+^-ATPase, CaMs, and CDPKs [61, 62]. Surprisingly, none of Ca^2+^ sensors from the CBL family showed up-regulated in both samples although most of its target kinases (e.g., CIPK10 homolog CL6537.contig1, CIPK6 homolog unigene17612, CIPK11 homolog unigene35076, and CIPK5 homolog unigene29689) were up-regulated. Given that the overexpression of CBL1 could result in attenuation of CBF/DREB induction [22], we speculate that the negative regulatory mechanism of *CBL* in cold perception may exist in *P. euphratica.* The complex cold responsive network composed of CBL calcium sensors and their target kinases require detailed analysis at the molecular level. Our results indicate that Ca^2+^ binding proteins are one of the signaling components induced at early stages of low temperature stress.

## Conclusions

*P. euphratica* is a perennial desert tree with a high capacity for low temperature resistance compared with other poplar species [63]. Here, we profiled the *P. euphratica* transcriptome under low temperature treatments in order to understand cold perception in this species. We obtained 108,502 assembled unigenes using the Trinity de novo assembly method and identified numerous potential cold sensing transcription factor genes and various key signal transduction components at the transcriptome level. These data provide useful resources for gene mining to improve low temperature stress tolerance in plants. Our results indicate not only that the CBF orthologs play key roles, but also that unsaturated fatty acids, ABA and calcium signaling components underlie a rapid and flexible cold response mechanism in *P. euphratica*. Compared with transient chilling stress response, fewer genes were found to have altered expression under temperature below freezing point. Particularly, only 3 pathways (*q* ≤ 0.05) clustered significantly with DGEs under freezing treatment. Taken together, our results provide valuable information on the molecular adaptation of *P. euphratica* to low temperature stress.

## Methods

### Stress treatment and RNA isolation

Two year-old *P. euphratica* seedlings, obtained from the Xinjiang Autonomous Region of China, were planted in individual pots (15 L) containing loam soil and placed in a greenhouse at Beijing Forestry University. Each pot contained four individuals. The temperature in the greenhouse was 22°C – 25°C with a 16 hour photoperiod (110–150 μmol.m^-2^.s^-1^). Potted plants were irrigated at a three-day interval based on evaporation demand for two months before treatment. In the chilling stress treatment group, *P. euphratica* plants were subjected to 4°C temperature under weak light. Simultaneously, some *P. euphratica* plants were subjected to a further drop of 8°C (-4°C) temperature under weak light conditions. The samples were harvested 6 h later and stored at -80°C until RNA extraction. Untreated *P. euphratica* plants harvested at 22°C under weak light was used as controls.

Total RNA was extracted from leaves using the CTAB method [64], treated with RNAase free DNAase. The A260/A280 ratios of the RNA samples were examined by NanoDrop 2000 and those with values ranging from 1.9 to 2.1 were chosen. The integrity of the RNA samples was examined with an Agilent 2100 Bioanalyzer. All RNA integrity number (RIN) values were over 8.0 and no signs of degradation were observed. Approximately 25 μg RNA sample with a concentration of ≥ 750 ng/μl from the CK, C4, and F4 samples were used for cDNA library construction.

### Illumina cDNA library preparation and sequencing

An Illumina Hiseq 2000 library was constructed for Solexa sequencing. Poly (A) mRNA was first enriched with oligo (dT), and then was fragmented into small pieces of 200–700 bp using divalent cations at an elevated temperature. Based on these cleaved RNA fragments, we used random hexamer-primer and reverse transcriptase (Invitrogen) to synthesize cDNA. Three paired-end cDNA libraries with an insert size of 200 bp were constructed, and then sequenced with the Illumina HiSeq™ 2000 to generate an average read length of 90 bp as raw data.

### De novo assembly and assessment

Raw data generated from Solexa sequencing were preprocessed to remove nonsense sequences including (1) adapters that were added for reverse transcription and sequencing, (2) sequences containing too many unknown bases (>5%), and (3) low-quality bases (>50% of the bases with a quality score ≤5). The preprocessed sequences were then assembled by Trinity [21] to construct contigs with default or optimal parameter. The contigs were then realigned to construct unigenes by Trinity. To fill the intra-scaffold gaps, we then used the paired-end information to retrieve read pairs that had one read well-aligned on the contigs and another read located in the gap region, and then locally assembled the collected reads. After gap closure, we constructed a non-redundant Unigene set from all three assembled datasets using the EST assembly program TGICL [65]. All clean reads were mapped back to the assembled transcripts using standalone Bowtie v0.12.8 and the percentage of reads that used in assembling was calculated. To decide the sequential orientation for each unigene, a set of sequential BLASTx searches against GenBank’s non-redundant database, the Swiss-Prot protein database (http://www.expasy.ch/sprot), the KEGG pathways database [66], and the COG database (http://www.ncbi.nlm.nih.gov/COG/) were carried out. The sequential orientation orders of the unigenes not found in any of the four databases were deciphered by ESTScan software [22].

To assess the quality of the de novo assembly, a similarity search against *P. trichocarpa* transcripts (version 3) was further conducted using BLASTN algorithm with E-value less than 1.0E-5. The unigenes with identities higher than 75% were listed. The *P. trichocarpa* transcripts (version 3) and annotated gene set were downloaded from the Department of Energy Joint Genome Institute (DOE-JGI; http:://www.phytozome.net/poplar). All unigenes were also compared with data in publicly available databases including 13,991 unassembled *P. euphratica* ESTs from GenBank and assembled ESTs from other poplar species in the TIGR Plant Transcript Assemblies database.

### Unigene annotation and function classification

To find the most descriptive annotation for each sequence, all assembled unique sequences were searched against NR, Swiss-Prot, KEGG, and COG using the BLASTx and against Nt using the BLASTn algorithms with a threshold of E < 1.0E-5. The protein with the highest sequence similarity was retrieved. For NR annotation, Blast2GO software [2] was first used to perform gene annotation, and then the WEGO and AGRIGO software [67] were used to conduct GO functional classification to interpret the distribution of gene functions defined by molecular function, cellular component, and biological process ontologies. Unigene sequences were also aligned to the COG database to predict and classify gene functions. Pathway assignments were carried out according to the KEGG pathway database using BLASTx with an E-value threshold of 1.0E-5.

### Protein-coding region prediction and transcription factor analysis

For protein coding sequence prediction, unigenes were searched against the NR, Swiss-Prot, KEGG, and COG protein databases in that order using a BLASTx algorithm (E-value < 1.0E-5). Unigenes that aligned to a high priority database were not aligned to databases of lower priority. The correct reading frame of the nucleotide sequences (5’-3’direction) of unigenes was defined by the highest rank in the BLAST results, and the corresponding protein sequences were obtained from the standard codon table.

Transcription factors were predicted according to protein sequences obtained from CDS prediction. We used hmmsearch to search for domains of the plant transcription factors (http://plntfdb.bio.uni-potsdam.de/v3.0/) and classified unigenes according to the gene family information. Genes that were believed to be associated with cold stress were selected for further investigation.

### Gene expression analysis

To identify genes regulated by low temperature stress, we measured gene expression levels by using numbers of fragments per kilobase of exon region in a given gene per million mapped fragments (FPKM) [68]. The FPKM method formula was:

where *C* is the number of reads that uniquely aligned to one unigene; *N* is the total number of reads that uniquely aligned to all unigenes; *L* is the base number in the CDS of one unigene. The goal of this transformation is to normalize the counts in regard of the differing library sizes and the length of the transcripts [69].

We identified DEGs from different samples (CK, C4 and F4) using R program. The Pearson’s chi-square test was applied to assess the lane effect. For each gene, the P-value was computed. After that, Benjamini–Hochberg false discovery rate (FDR) was applied to correct the results for *p* value. FDR method is widely used in deep-sequencing studies because of its power in finding over-representative unigenes [70–73]. The transcripts that were induced or suppressed at an estimated absolute log_2_-fold change of >1 and FDR adjusted *p*-value ≤ 0.05 were considered to be differentially expressed [74].

### GO and KEGG analyses for differentially expressed unigenes

In order to find the significantly enriched GO terms in DEGs against a genome background, the DEGs were annotated to GO database (http://www.geneontology.org/) using hypergeometric test for statistical analysis [25]. For *p* value correction, we used the rigorous Bonferroni correction method. The cutoff *p* value after correction was 0.05. GO terms fulfilling this condition were defined as being significantly enriched. The KEGG pathway enrichment analysis of DEGs was also performed with the whole genome background as a reference to find the main biochemical pathways and signal transduction pathways in which DEGs involved. After multiple testing corrections, we defined pathways with *q*-value ≤0.05 as being those significantly enriched in DEGs.

### Quantitative PCR analysis

Quantitative PCR (qPCR) was performed to determine the expression level of selected unigenes. The qPCR was conducted using a power SYBR Green PCR Kit (ABI) in a MicroAmp™ 96-well plate with a StepOnePlus™ Real-Time PCR System (ABI). The relative expression value was calculated by the 2^-ΔΔCt^ method using *PeActin* (GenBank accession number EF148840) as an internal control [75]. Gene-specific primers used in the qPCR analysis are listed in Additional file 14. RNA pools used in the qPCR analyses were extracted from three independent samples which were different from those used for RNA-seq. Three technical replicates were used for each sample.

## Electronic supplementary material

Additional file 1: **Overview of the number of reads that could be mapped back to assembled transcripts.** (XLSX 11 KB)

Additional file 2: **Assessment of the unigene assembly based on comparison to poplar ESTs.** All unique sequences generated from different assemblages were subjected to a BLAST comparison to compare EST collections from a variety of poplar species. (XLSX 11 KB)

Additional file 3: **Alignment against the** ***P. trichocarpa*** **transcripts with an** **E-value threshold of 1.0E-5 and identity threshold of 75%.** (XLSX 4 MB)

Additional file 4: **Functional annotation summary.** We aligned the unigene sequences to the Nr, Swiss-Prot, KEGG and COG databases by BLASTx (E-value < 0.00001) and to the nucleotide sequence database Nt (E-value < 0.00001) by BLASTn. We thus obtained proteins with the highest similarity to the given unigenes, as well as the functional annotations. (DOCX 16 KB)

Additional file 5: **100 most abundant transcripts in three** ***P. euphratica*** **sample sets.** (DOCX 18 KB)

Additional file 6: **Differentially expressed genes among three samples.** Unigenes that expressed differentially in at least two samples were list in this file. (XLSX 359 KB)

Additional file 7: **Transcripts that differentially expressed in all three samples.** Unigenes that expressed differentially in all three samples were list in this file. (XLSX 29 KB)

Additional file 8: **Differentially expressed genes between CK and C4.** (XLSX 198 KB)

Additional file 9: **Differentially expressed genes between CK and F4.** (XLSX 103 KB)

Additional file 10: **Differentially expressed genes between C4 and F4.** (XLSX 149 KB)

Additional file 11: **Clustering of the top 50 well-annotated genes that are up-regulated in C4.** All unigenes were annotated by NR or *P. trichocarpa* version 3 annotation dataset. (XLSX 33 KB)

Additional file 12: **Clustering of the top 50 well-annotated genes that are up-regulated in F4.** All unigenes were annotated by NR or *P. trichocarpa* version 3 annotation dataset. (XLSX 15 KB)

Additional file 13: **Pathway enrichment analyses in DEGs (** ***q*** **value ≤ 0.05).** (XLSX 11 KB)

Additional file 14: **Primer sequences for qPCR.** The primers used in quantitative real-time PCR analysis. (DOCX 18 KB)

## Abbreviations

COR: Cold-responsive
CBF: CRT binding transcription factor
RNA-seq: RNA sequencing
DEG: Differentially expressed gene
FDR: False discovery rate
FPKM: Fragments per kilobase of exon region in a given gene per million mapped fragments
GO: Gene ontology
KEGG: Kyoto Encyclopedia of Genes and Genomes
qPCR: Quantitative real-time PCR
TF: Transcription factor.
