# Supplementary material for: Deep-sequencing transcriptome analysis of low temperature perception in a desert tree, Populus euphratica
Source: BMC Genomics. 2014 May 1;15(1):326. doi: 10.1186/1471-2164-15-326 (PMC4035058; doi:10.1186/1471-2164-15-326)
Supplement: Supplementary file 4 — Additional file 4: Functional annotation summary. We aligned the unigene sequences to the Nr, Swiss-Prot, KEGG and COG databases by BLASTx (E-value < 0.00001) and to the nucleotide sequence database Nt (E-value < 0.00001) by BLASTn. We thus obtained proteins with the highest similarity to the given unigenes, as well as the functional annotations. (DOCX 16 KB) [file 12864_2013_6038_MOESM4_ESM.docx]

**Additional file 4 Functional annotation summary**

|  | **Database** | **Number** | **Percent（%）** |
| --- | --- | --- | --- |
| **Annotated** | NR | 71,428 | 75.83 |
|  | NT | 84,932 | 90.17 |
|  | SwissProt | 42,396 | 45.01 |
|  | KEGG | 39,313 | 41.74 |
|  | COG | 25,675 | 27.26 |
|  | GO | 39,652 | 42.10 |
|  | Total | 85,584 | 90.86 |
| **Unannotated** |  | 8612 | 9.14 |
