# Supplementary material for: Deep-sequencing transcriptome analysis of low temperature perception in a desert tree, Populus euphratica
Source: BMC Genomics. 2014 May 1;15(1):326. doi: 10.1186/1471-2164-15-326 (PMC4035058; doi:10.1186/1471-2164-15-326)
Supplement: Supplementary file 5 — Additional file 5: 100 most abundant transcripts in three P. euphratica sample sets. (DOCX 18 KB) [file 12864_2013_6038_MOESM5_ESM.docx]

**Additional file 5 100 most abundant transcripts in three *P. euphratica* sample sets**

|  | **Number** | **Gene list** |
| --- | --- | --- |
| **Common genes of three samples** | 68 | Unigene32644;Unigene36719;Unigene17190;Unigene30841;CL4767.Contig1;Unigene6057;Unigene27567;Unigene9736;CL8018.Contig1;Unigene11847;CL8394.Contig3;CL1588.Contig1;CL2303.Contig2;CL419.Contig3;CL6169.Contig1;Unigene9604;CL7481.Contig2;CL662.Contig1;Unigene5912;CL6772.Contig1;CL5710.Contig1;CL6772.Contig2;Unigene23502;Unigene8075;Unigene13549;Unigene13465;CL6538.Contig2;Unigene25082;Unigene11189;CL7481.Contig1;CL419.Contig2;CL2549.Contig4;CL2332.Contig1;CL8176.Contig1;Unigene17421;CL7438.Contig2;CL10374.Contig4;Unigene25057;CL3677.Contig4;Unigene31432;Unigene11924;CL13705.Contig1;Unigene2077;Unigene46021;Unigene37037;CL5623.Contig1;Unigene21623;CL14216.Contig3;CL10213.Contig1;CL2426.Contig2;CL5150.Contig1;CL12531.Contig2;CL1764.Contig1;CL8736.Contig2;Unigene32738; Unigene13530; Unigene35320; Unigene13953; CL5642.Contig1; CL2493.Contig1;CL6659.Contig1;Unigene23887;CL825.Contig1;Unigene13918;CL2493.Contig4;Unigene17240;Unigene23160;CL12378.Contig2 |
| **Common genes of C4 and CK** | 9 | CL1933.Contig9;CL12124.Contig2;Unigene28762;CL2911.Contig10;CL1284.Contig5;Unigene13916;CL1517.Contig8;CL164.Contig2;Unigene12084 |
| **Common genes of C4 and F4** | 4 | Unigene30842;CL9698.Contig1;Unigene13906;Unigene860;CL4391.Contig1 |
| **CK Unique genes** | 23 | CL1114.Contig1;CL6705.Contig1;CL6953.Contig1;CL11487.Contig1;CL13383.Contig1;CL8263.Contig1;CL10819.Contig1;Unigene17369;Unigene31336;CL4664.Contig1;CL5206.Contig2;Unigene2577;CL8727.Contig2;Unigene2302;Unigene9693;Unigene15687;CL11526.Contig3;Unigene5925;CL2057.Contig2;CL5160.Contig2;CL2057.Contig1;CL2740.Contig1;CL11632.Contig1 |
| **C4 unique genes** | 19 | CL10821.Contig2;CL11971.Contig1;CL13025.Contig2; Unigene5939; Unigene22971;CL7896.Contig3; CL419.Contig12; CL5180.Contig1; Unigene36500; Unigene17714;Unigene2586; CL2197.Contig1; CL5782.Contig1; CL13013.Contig1; Unigene13473; CL6772.Contig3; CL2040.Contig1; CL522.Contig9 |
| **F4 Unique genes** | 28 | CL11474.Contig1;Unigene9693;CL2493.Contig2;Unigene24953;CL5160.Contig2;Unigene17351;Unigene7861;Unigene17369;Unigene31336;CL11487.Contig1;CL8248.Contig3;CL3921.Contig2;Unigene15687;Unigene2577;CL4664.Contig1;CL13383.Contig1;Unigene36156;Unigene29019;Unigene2302;CL11526.Contig3;Unigene15688;CL2740.Contig1;CL4126.Contig12;CL8263.Contig1;CL6705.Contig1;CL13351.Contig2;CL2057.Contig1 |
