# Supplementary material for: Deep-sequencing transcriptome analysis of low temperature perception in a desert tree, Populus euphratica
Source: BMC Genomics. 2014 May 1;15(1):326. doi: 10.1186/1471-2164-15-326 (PMC4035058; doi:10.1186/1471-2164-15-326)
Supplement: Supplementary file 14 — Additional file 14: Primer sequences for qPCR. The primers used in quantitative real-time PCR analysis. (DOCX 18 KB) [file 12864_2013_6038_MOESM14_ESM.docx]

**Additional File 14 Primer sequences for qPCR**

| Gene ID | Primer name | Sequence (5’-3’) |
| --- | --- | --- |
| *Unigene50330* | 30F | AACTTCTTCTTGCCAACTGG |
|  | 30R | GCTTTGATTTCTTCTGCCG |
| *Unigene50527* | 27F | CCATACGACCTGCTCCT |
|  | 27R | GCTTGAAAGTGGAAATGC |
| *Unigene50887* | 87F | TTATTTCCTCAGAAGTCTCAGC |
|  | 87R | CACCGCCATTGTTGTATC |
| *Unigene26311* | 311F | ACGCCAAAGCAGCAACAT |
|  | 311R | GGGAAACAAGGCATCCTG |
| *Unigene26567* | 67F | GGAATGGCGATAAATGGG |
|  | 67R | CAACTCTGCTGCCTCACTTG |
| *Unigene18620* | 20F | AACAAGAACACCAACACCAT |
|  | 20R | GAAATCACTCAAGTTCAGGTG |
| *Unigene28779* | 79F | AGGGCGAAGCACAAATCT |
|  | 79R | GCGGTGTCTACTTTCACAGTT |
| *Unigene18453* | 53F | CAGTGTTCTCATTCTCCTGTTC |
|  | 53R | GATGCCTTGTTTCCCTGA |
| *Unigene17351* | 51F | CAACGGAGGAAGAATACCTTG |
|  | 51R | GGTGGACTCAGAGGAGGATT |
| *Unigene7110* | 110F | TCCTCTTCAGGCTGGTAGG |
|  | 110R | GGATGGAGATGGGTTGTTG |
